# Evaluation of mitochondrial biogenesis and ROS generation in high-grade serous ovarian cancer

**DOI:** 10.3389/fonc.2023.1129352

**Published:** 2023-03-01

**Authors:** Zeynep C. Koc, Vincent E. Sollars, Nadim Bou Zgheib, Gary O. Rankin, Emine C. Koc

**Affiliations:** ^1^ Department of Obstetrics, Gynecology and Reproductive Sciences, Temple University, Philadelphia, PA, United States; ^2^ Department of Biomedical Sciences, Joan C. Edwards School of Medicine, Marshall University, Huntington, WV, United States; ^3^ Edwards Comprehensive Cancer Center, Joan C. Edwards School of Medicine, Marshall University, Huntington, WV, United States

**Keywords:** mitochondrial biogenesis, mitochondrial translation and transcription, mitochondrial reactive oxygen species (mtROS), oxidative phosphorylation (OXPHOS), high-grade serous ovarian cancer (HGSOC), MT-COII, TFAM, TUFM

## Abstract

**Introduction:**

Ovarian cancer is one of the leading causes of death for women with cancer worldwide. Energy requirements for tumor growth in epithelial high-grade serous ovarian cancer (HGSOC) are fulfilled by a combination of aerobic glycolysis and oxidative phosphorylation (OXPHOS). Although reduced OXPHOS activity has emerged as one of the significant contributors to tumor aggressiveness and chemoresistance, up-regulation of mitochondrial antioxidant capacity is required for matrix detachment and colonization into the peritoneal cavity to form malignant ascites in HGSOC patients. However, limited information is available about the mitochondrial biogenesis regulating OXPHOS capacity and generation of mitochondrial reactive oxygen species (mtROS) in HGSOC.

**Methods:**

To evaluate the modulation of OXPHOS in HGSOC tumor samples and ovarian cancer cell lines, we performed proteomic analyses of proteins involved in mitochondrial energy metabolism and biogenesis and formation of mtROS by immunoblotting and flow cytometry, respectively.

**Results and discussion:**

We determined that the increased steady-state expression levels of mitochondrial- and nuclear-encoded OXPHOS subunits were associated with increased mitochondrial biogenesis in HGSOC tumors and ovarian cancer cell lines. The more prominent increase in MT-COII expression was in agreement with significant increase in mitochondrial translation factors, TUFM and DARS2. On the other hand, the ovarian cancer cell lines with reduced OXPHOS subunit expression and mitochondrial translation generated the highest levels of mtROS and significantly reduced SOD2 expression. Evaluation of mitochondrial biogenesis suggested that therapies directed against mitochondrial targets, such as those involved in transcription and translation machineries, should be considered in addition to the conventional chemotherapies in HGSOC treatment.

## 1 Introduction

Ovarian cancer is one of the deadliest gynecological cancers worldwide and is the fifth leading cause of death for women in the United States (1). Despite success in attaining remission in many cases, over half of the women with ovarian cancer experience resistance to chemotherapy, metastasis, and recurrence. Changes in energy and antioxidant metabolism have been highlighted as major factors in chemoresistance and peritoneal metastasis in recent epithelial high-grade serous ovarian cancer (HGSOC) studies (2–6). Determining the metabolic remodeling of energy generation for metastatic development and tumor growth has the potential to introduce pathway-specific therapies.

In recent biomarker studies, mitochondrial energy metabolism is emerging as one of the major contributors to aggressiveness and chemoresistance in HGSOC (7, 8). The mitochondrial mass and oxidative phosphorylation (OXPHOS) capacities are increased 3.3-8.4-fold in epithelial ovarian carcinoma (9). It is believed that tumors preferentially use aerobic glycolysis rather than the much more efficient OXPHOS to generate ATP, described as the Warburg effect (10–12). However, evidence suggests that tumor cells require a metabolically rich microenvironment allowing a combination of aerobic glycolysis and OXPHOS to promote growth and metastasis (13, 14). In addition to increased OXPHOS, high levels of reactive oxygen species (ROS) generated in HGSOC cause sensitivity to platinum-based chemotherapy (7, 15). However, HGSOC tumors have been shown to develop resistance to platinum-based chemotherapy over time, possibly due to remodeling of the energy metabolism and apoptotic pathways (5, 7, 16–18).

The metabolic flexibility of HGSOC tumors requires changes in the expression of both nuclear and mitochondrial genomes to encode subunits of OXPHOS complexes (complex I-V). Mitochondrial transcription supports the synthesis of 13 OXPHOS subunits encoded by the mitochondrial genome, two ribosomal RNAs (rRNAs), and 22 tRNAs (19, 20). Malignant transformation of mitochondrial function and mtDNA mutations have been observed in age-related cancer development (21, 22). A comprehensive list of mitochondrial genes and proteins causing mitochondrial dysfunction in ovarian cancer can be found in a recent review published by Shukla and Singh (23).

The significant variation in the expression of mitochondrial-specific transcription factors, such as PGC1α and TFAM, implies a highly modulated expression of mt-transcription in HGSOC (9, 24–26). Activation of PGC1α, promoted by chronic oxidative stress and aggregation of PML-nuclear bodies, results in high OXPHOS capacity and chemosensitivity in HGSOC (7). On the other hand, the knock-down of PGC1α or TFAM diminishes the generation of mitochondrial reactive oxygen species (mtROS) and cisplatin-induced apoptosis (27).

The role of mitochondrial translation in HGSOC is limited. Nuclear-encoded protein factors and mitochondrial-specific 55S ribosomes support mitochondrial translation. While the mitochondrial ribosomal proteins (MRPs) are all nuclear-encoded genes, 55S ribosomes are composed of the two mitochondrial(mt)-encoded rRNAs and 80 MRPs identified in our previous proteomics studies (28–32). For the mitochondrial translation-related genes, high transcript levels of mitochondrial ribosomal small (MRPS) and large (MRPL) subunit proteins, MRPS12, MRPS14, MRPL15, MRPL34, and MRPL49, are suggested as novel prognostic markers predicting reduced overall survival in ovarian cancer patients (33–35). Additionally, a single nucleotide polymorphism (SNP) of the mitochondrial elongation factor Tu (TUFM) gene is associated with epithelial ovarian cancer risk (36). Therefore, further evaluation of factors involved in mitochondrial biogenesis, specifically mitochondrial translation, is required to determine the mechanism(s) behind the remodeling of energy metabolism in HGSOC and resistance to chemotherapy.

Here, we provide evidence, for the first time, that changes in mitochondrial biogenesis support the metabolic flexibility in HGSOC tumor biopsies and ovarian cancer cell lines. Specifically, mitochondrial translation and transcription factors played an essential role in the modulation of OXPHOS subunit expression. Datamining analyses of mass spectrometry (MS)-based proteomics studies of HGSOC performed by the Clinical Proteomic Tumor Analysis Consortium (CPTAC) and Institute Curie cohort also supported our findings with concurrent changes in mt-encoded subunit II of the complex IV, MT-COII, and mitochondrial translation factors, TUFM and DARS2. We also observed higher mtROS generation in ovarian cancer cell lines with lower OXPHOS subunit expression and mitochondrial biogenesis. These observations suggest that the steady-state expression of mt-encoded OXPHOS subunits and components of the mitochondrial translation could be used as prognostic biomarkers to determine more targeted chemotherapy options in HGSOC.

## 2 Materials and methods

### 2.1 Ovarian tissue biopsies

Fifteen de-identified ovarian tumors and normal tissue biopsies were removed by surgical excision from patients treated at the Marshall University Edwards Comprehensive Cancer Center, Huntington, WV. Ethical review and approval were not required for the human de-identified biopsies used in this study in accordance with the local legislation and institutional requirements. Tumor characteristics of biopsies are given in **Table S1**. Ovarian cancer subtypes were determined by immunohistochemistry, immunofluorescence, and fluorescence *in situ* hybridization techniques by the Edwards Comprehensive Cancer Center. Tissue protein lysates were prepared by resuspension and sonication of biopsies in RIPA buffer containing 1% SDS and NP40. Protein concentration was determined by the bicinchoninic acid (BCA) assay (Pierce, Rockford, USA).

### 2.2 Cell culture and [^35^S]-Met pulse labeling

The NCI-ovarian cancer cell line panel (OVCAR-4, OVCAR-5, OVCAR-8, SKOV-3, and IGROV-1) was purchased from NCI. Using gene expression compositional assignment, the OVCAR-5 cell line is also reported as being gastrointestinal in origin (37); however, NCI did not confirm this report. The OVCAR-3 cell line was obtained from Dr. Sarah Miles (Marshall University). The NCI-60 panel of ovarian cancer cell lines, OVCAR-4, OVCAR-5, OVCAR-8, SKOV-3, and IGROV-1, was cultured in RPMI media (HyClone, Thermo-Scientific, Waltham, MA) as recommended by NCI. OVCAR3 cells were maintained in RPMI media containing 20% fetal bovine serum (FBS) (Rocky Mountain Biologicals, Missoula, MT), 10 mL/mL human insulin, 0.1% penicillin/streptomycin (Corning Cellgro, Manassas, VA). The cells were grown in a humidified incubator at 37°C with 5% CO_2_. All experiments with the cell lines were limited to passages 5-15 from frozen stocks and repeated with a minimum of nine biological replicates conducted in three separate experiments for all results.

Expression of the 13 mt-encoded subunits of OXPHOS complexes was determined by [^35^S]-Met pulse labeling described previously (38, 39). Pulse labeling experiments were performed with breast cancer cell lines grown to 60-70% confluency in RPMI media. After arresting cytosolic protein synthesis by emetine, cells were incubated in 0.2 mCi/mL of [^35^S]-EasyTag™ Protein Labeling Mix (Perkin Elmer Inc., Waltham, MA) containing media for 2 h. Cells were lysed in RIPA buffer supplemented with protease and phosphatase inhibitors (Calbiochem, Darmstadt, Germany). Whole-cell lysates (30 μg) were separated on 13% SDS-PAGE. The gels were dried on 3MM chromatography paper (Whatman) after Coomassie Blue staining, and the signal intensities of the bands were quantified by UN-Scan-It (Silk Scientific Inc, Orem, UT).

### 2.3 Immunoblotting analyses

Tissue lysates obtained from biopsies and cell lines were either diluted further or lysed in RIPA buffer containing 50 mM Tris-HCl (pH 7.6), 150 mM NaCl, 1 mM EDTA, 1 mM EGTA, 1% NP40, 0.1% SDS, 0.5% deoxycholate, and protease and phosphatase inhibitor cocktails (Calbiochem, Darmstadt, Germany). Protein concentrations were determined using BCA assays (Pierce, Rockford, USA). Approximately 20 µg of the protein lysate was separated on 12% SDS-PAGE, transferred to nitrocellulose membranes (Amersham, GE Healthcare, UK), and stained with Ponceau S to ensure equal protein loading (**Figures S1, S2**). The Ponceau S staining of nitrocellulose membranes was used to normalize total protein loading to signal intensities detected by immunoblotting analyses. Antibodies were commercially obtained as follows: the human OXPHOS antibody cocktail from Abcam (Eugene, OR); mitochondrial NDUSF2, DARS2, TUFM, and TFAM from Santa Cruz (Dallas, TX); PGC1α and SSBP1 from ProteinTech (Rosemont, IL), SOD2 from Cell Signaling Technologies (Danvers, MA), and GAPDH from Fitzgerald (Acton, MA). The secondary anti-rabbit and mouse HRP-conjugate antibodies were obtained from Pierce (Rockford, USA). The protein immunoreactivity was detected using the ECL Western blotting kit (Amersham, GE Healthcare, UK) as directed by the manufacturer. Immunoblotting signal intensities were quantified by UN-Scan-It (Silk Scientific Inc, Orem, UT) and normalized to total protein loading detected by Ponceau S staining of the membranes.

### 2.4 Flow cytometry analyses

Mitochondrial mass and ROS generation determinations in ovarian cancer cell lines were performed using MitoTracker-Red CMXRos (Invitrogen) and MitoSOX-Red (ThermoFisher), respectively, by flow cytometry analyses using the Agilent Novocyte 3000. Data analysis was performed using NovoExpress Software vX. Optimal concentrations of Mito-SOX-Red and Mito-Tracker-Red were 5 and 0.5 μM, respectively.

### 2.5 Statistical analyses

Statistical and graphical analyses were performed using Excel and GraphPad Prism 9.3. Statistical significance was determined using unpaired Welch’s *t*‐tests. Probability values less than 0.05 were regarded as statistically significant. All the values were in triplicates wherever possible and expressed as the mean ± SD unless otherwise described.

## 3 Results and discussion

### 3.1 Heterogeneity of mitochondrial energy metabolism in ovarian cancer

Changes in mitochondrial energy metabolism have recently been suggested as possible causes for chemoresistance and tumor recurrence in HGSOC (2, 7, 23, 40, 41). To further investigate the changes in mitochondrial function, we obtained nine surgically removed normal ovarian and ovarian tumor tissue biopsies from the Tissue Procurement Center at the Marshall University Edwards Comprehensive Cancer Center, Huntington, WV. The tumor characteristics and stages of HGSOC biopsies are given in the **Supplemental Table S1**.

**Figure 1:**
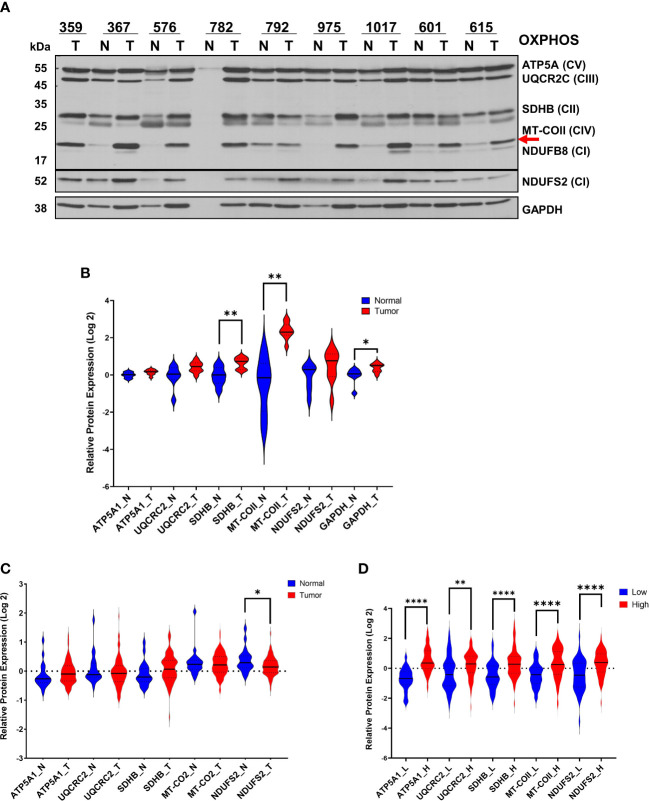
Modulation of OXPHOS subunit expression in ovarian cancer. **(A)** The expression of OXPHOS subunits, including ATP5A1 (CV; Complex V), UQCRC2 (CIII; Complex III), SDHB (CII, Complex II), MT-COII (CIV; Complex IV), NDUFB8 and NDUFS2 (CI; Complex I), and GAPDH were detected by immunoblotting in normal **(N; blue)** and ovarian tumor **(T; red)** biopsies. Only a tumor biopsy was available for patient 359. Approximately 20 μg of protein lysates obtained from normal and tumor tissues were separated by 12% SDS-PAGE, and the immunoblotting analyses were performed using antibodies shown on the left side. For patient 782, the normal tissue protein amount was extremely low, and it was not included in the quantitation. The red arrow shows the mt-encoded complex IV subunit, MT-COII. **(B)** Relative protein expression was quantified by normalizing the signal intensities of antibodies to protein loading and presented as violin graphs after conversion to log 2 values. **(C)** Log2 protein expression values of OXPHOS subunit expression determined by MS-based proteomics of 20 normal (N; blue) and 83 HGSOC ovarian biopsies **(T)** published by McDermott et al. [Log 2 values are taken from **Supplemental Data** in reference (45)] as part of the CPTAC. **(D)** Log2 OXPHOS subunit expression values for 53 low (L; blue) and 74 high (H; red) HGSOC tumors were reported by Gentric et al. [Log 2 values are taken from **Supplemental Data** in reference (7)] as part of the Curie cohort. For the statistical analysis, unpaired Welch’s t-test was used, and the P values ≤ 0.05 were represented as (*), ≤ 0.01 (**) and ≤ 0.0001 (****).

In our earlier studies, the steady-state expression of OXPHOS subunits by immunoblotting agrees with OXPHOS complex activities and is sufficient for evaluating energy metabolism in mitochondria (7, 42–44). Therefore, we determined the steady-state expression of OXPHOS subunits in HGSOC biopsies by immunoblotting using an antibody cocktail. The antibody cocktail is a mixture of five antibodies recognizing four nuclear-encoded OXPHOS subunits, including complex V (ATP5A1), III (UQCRC2), II (SDHB), and I (NDUFB8) and a mt-encoded subunit of complex IV (MT-COII). The same OXPHOS membrane was also probed with GAPDH antibody (**Figure 1A**). The signal intensity detected by NDUFB8 antibody would not be quantified in most of the biopsies. Instead, an additional antibody against NDUFS2 was used to confirm changes in another nuclear-encoded subunit of complex I (**Figure 1A**). Signal intensities obtained for each subunit were normalized to total protein loading detected by Ponceau S staining and mean signal intensities for normal and tumor biopsies rather than a direct comparison of normal and tumor biopsies from the same patient (**Figure 1B**). Changes in OXPHOS subunit expressions were at least 2-3-fold higher for some of the subunits in tumor biopsies relative to the normal tissue biopsies obtained from the same patient (**Figures 1A, B**). Specifically, we observed an overall statistically significant increase in complex II and IV subunits, SDHB and MT-COII (P< 0.01), as well as GAPDH in tumors relative to the normal tissues (**Figures 1A, B**). Although GAPDH is usually used as a loading control, here we observed an increase in the glycolytic enzyme, GAPDH (P<0.05), confirming the metabolic remodeling in HGSOC (10).

Recent proteomics data published by CPTAC has provided a more comprehensive MS-based quantitation of 20 normal and 83 ovarian tumor biopsies obtained from HGSOC patients (45). The Log2 expression values for mitochondrial proteins are reported in **Supplemental Data** provided by McDermott et al. (45). The majority of the OXPHOS subunit expression slightly increased in tumor biopsies relative to the normal tissues; however, NDUFS2 expression is reduced in tumor biopsies (p<0.05) (**Figure 1C**). The HGSOC proteome analyses by CPTAC compares 83 HGSOC tumor tissues to the mean values determined from 20 normal tissues (**Figure 1B**). Here, the slight discrepancy in the magnitude of expression changes in our results compared to those by CPTAC is possibly due to the direct comparison of the normal and tumor tissues obtained from the same patient in our analyses rather than a comparison to a mean as performed by CPTAC. However, when Gentric et al. compared the OXPHOS subunit expression by MS-based proteomics, HGSOC biopsies were classified as low- and high- OXPHOS subunit expressing HGSOC tumors (7). The expression of nuclear-encoded subunits ATP5A1, UCQRC2, SDHB, and NDUFS2, and the mt-encoded subunits, including MT-COII, were graphed to demonstrate a significant increase in OXHOS subunits in HGSOC tumors with high mitochondrial energy metabolism (**Figure 1D**). The agreement observed between our immunoblotting analyses and MS-based proteomics data by McDermott et al. and Gentric et al. suggests that the modulation of mitochondrial energy metabolism is required for tumor growth and proliferation in HGSOC (7, 45).

### 3.2 Mitochondrial biogenesis modulates OXPHOS in HGSOC

The up-regulation of mt-encoded MT-COII protein expression shown above (**Figure 1A**) indicates a role for mitochondrial biogenesis in HGSOC. Due to the presence of seven mt-encoded subunits in complex I, the nuclear-encoded complex I subunit, NDUFS2, was also concurrently affected by the changes in mitochondrial biogenesis (**Figure 1A**). However, the overall increase in NDUFS2 expression was not statistically significant in tissue biopsies (**Figure 1B**). Additionally, the transcription factors involved in nuclear- and mt-encoded OXPHOS transcripts, PGC1α and TFAM, respectively, are suggested as putative markers of chemoresistance in epithelial ovarian carcinoma (7, 25, 46). Therefore, we postulated that the mitochondrial transcription and translation proteins directly related to the biogenesis of 13 mt-encoded subunits also contribute to the modulation of OXPHOS in HGSOC.

**Figure 2:**
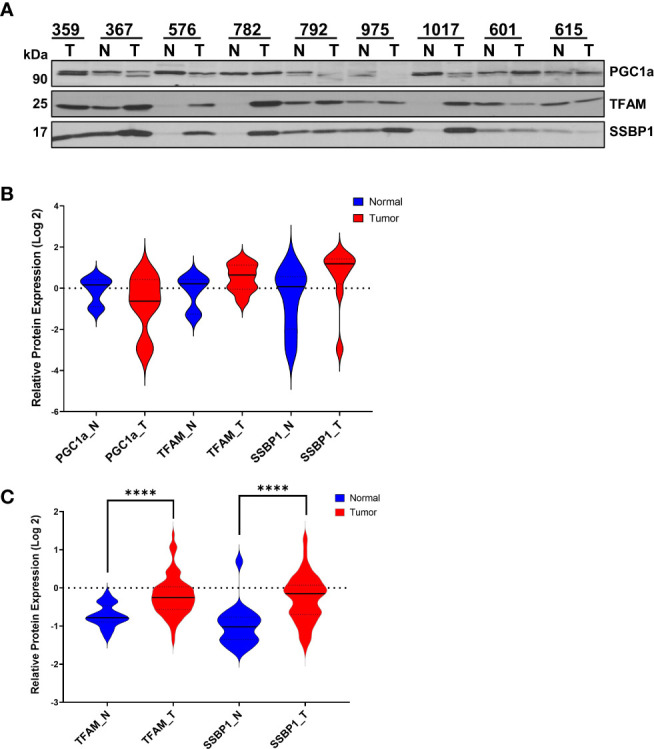
Expression of Mitochondrial Transcription- and Replication-Related Proteins in HGSOC. **(A)** Expression of PGC1α, TFAM, and SSBP1 proteins were detected in normal and HGSOC biopsies by immunoblotting analyses as described in **Figure1A**. **(B)** Log 2 relative protein expression values of PGC1a, TFAM, and SSBP1 shown in **(A)** were presented using violin plots. **(C)** MS-based quantitation of TFAM and SSBP1 published by McDermott et al. (45) as part of the CPTAC data set described in **Figure 1C**. The normal (N; blue) and HGSOC biopsies (T; red) were compared using violin plots as described in **Figure 1B** legend. P values ≤ 0.0001 were represented as (****).

To assess the role of mitochondrial biogenesis in HGSOC biopsies, we determined PGC1α and TFAM protein expression and the single-stranded mitochondrial DNA-binding protein (SSBP1) by immunoblotting using the same HGSOC biopsies described above (**Figures 2A**, **S2**). Although the PGC1α levels were slightly decreased and a doublet observed, TFAM and SSBP1 protein expression were elevated in some of the HGSOC tumor biopsies relative to the normal values (**Figure 2A**). The overall mean modulation of protein expression in normal *vs.* tumor biopsies was not significant (P>0.05) (**Figure 2B**). On the other hand, TFAM and SSBP1 levels were significantly elevated in the HGSOC tumor biopsies reported by the CPTAC proteome (P<0.0001) (**Figure 2C**) (45). These findings supported the altered mitochondrial transcription and replication in HGSOC.

**Figure 3:**
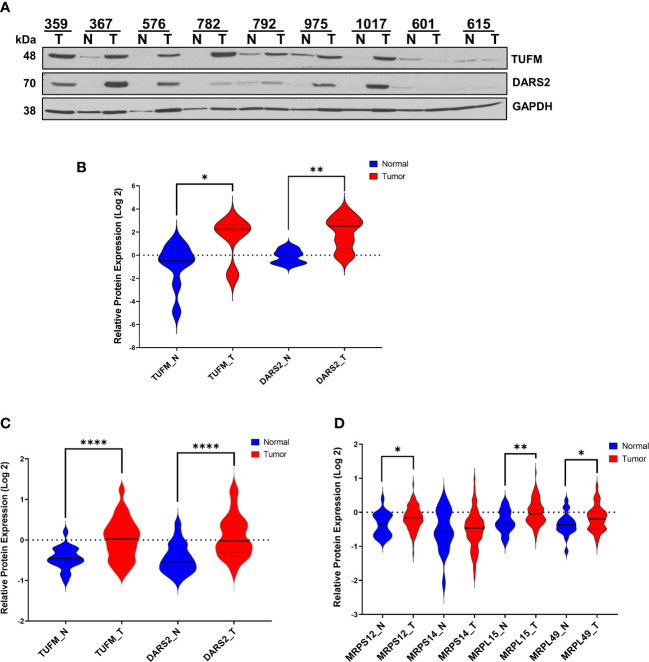
Expression of Mitochondrial Translation-Related Proteins in HGSOC. **(A)** Relative protein expression of TUFM and DARS2 were detected in normal ovarian **(N)** and HGSOC biopsies **(T)** by immunoblotting analyses. Equal protein loading was evaluated by Ponceau S staining and GAPDH probing. **(B)** Log 2 relative protein expression values of TUFM and DARS2 shown in panel A was presented using violin plots. **(C)** MS-based quantitation of TUFM and DARS2 protein expression, and **(D)** expression of mitochondrial ribosomal proteins, MRPS12, MRPS14, MRPL15, and MRPL49 in normal (N; blue) and HGSOC (T; red) in HGSOC published by McDermott et al. as part of the CPTAC data set [Log 2 values are taken from **Supplemental Data** in reference (45)]. P values ≤ 0.05 were represented as (*), ≤ 0.01 (**), and ≤ 0.0001 (****).

The modulation of OXPHOS requires cooperation between mitochondrial transcription and translation for synthesizing 13 mt-encoded subunits. We next determined the expression of two mitochondrial translation factors, elongation factor Tu (TUFM) and aspartyl-tRNA synthetase 2 (DARS2), in HGSOC biopsies by immunoblotting. Interestingly, the TUFM and DARS2 protein expressions were much higher and significant, P<0.05 and P<0.005, respectively, in tumor biopsies (**Figures 3A, B**). The CPTAC proteomics data mining analyses for TUFM and DARS2 were also in agreement with our observation (**Figure 3C**). In fact, the majority of mitochondrial translation-related proteins and factors are higher in the HGSOC tumor biopsies reported by the CPTAC (data not shown (45)).

Higher expression of several MRP genes has been associated with reduced overall survival and tumor recurrence using the publicly available ovarian cancer transcriptomics databases (33, 34). We searched the CPTAC proteome data to determine the expression of these MRPs, including MRPS12, MRPS14, MRPL15, and MRPL49, published by McDermott et al. (45). Log2 protein expression values for MRPS12, MRP L15, and MRPL49 were graphed and shown to be significantly elevated in tumor biopsies (**Figure 3D**). Here, the synergy between the MRP expression and mitochondrial translation factors along with the MT-COII expression confirmed the remodeling of energy metabolism and mitochondrial biogenesis, particularly the protein synthesis, in HGSOC (**Figures 1A**, **3**).

### 3.3 mtROS generation is increased in ovarian cancer cell lines with reduced OXPHOS subunit expression

**Figure 4:**
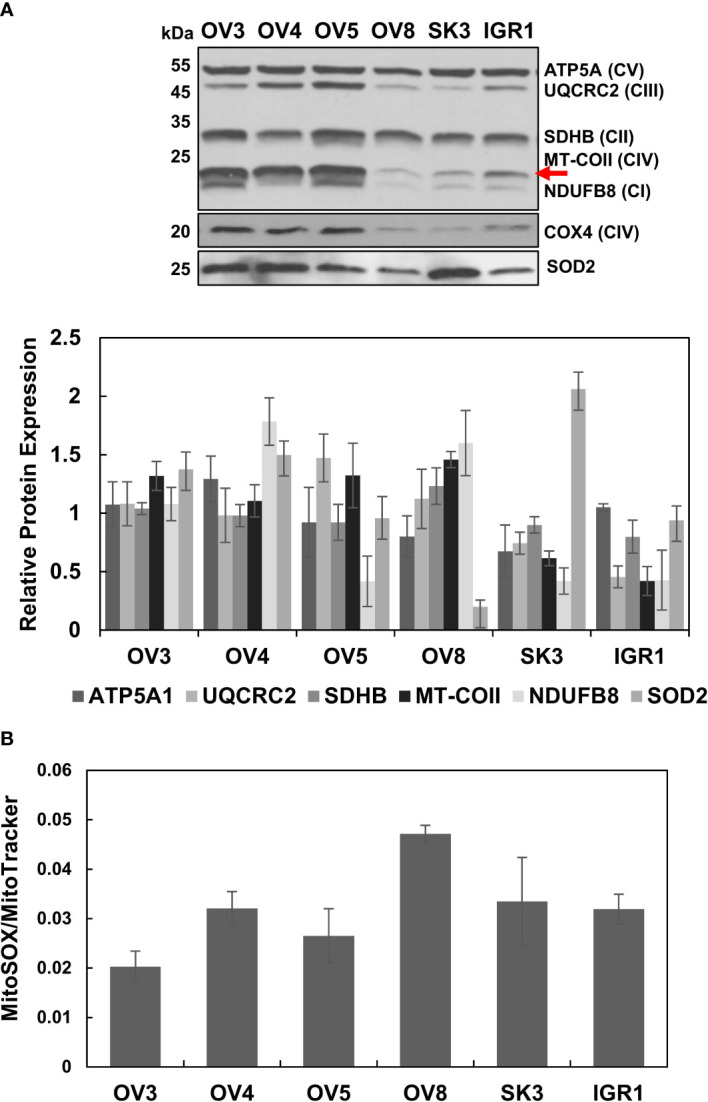
Altered OXPHOS subunit and SOD2 expressions and generation of reactive oxygen species in ovarian cancer cell lines. **(A)** The OXPHOS subunit and SOD2 expressions were detected by immunoblotting of lysates obtained from ovarian cancer cell lines, OVCAR-3 (OV3), OVCAR-4 (OV4), OVCAR-5 (OV5), OVCAR-8 (OV8), SKOV-3 (SK3), and IGROV-1 (IGR1) as described in **Figure 1A**. The red arrow shows the mt-encoded complex IV subunit, MT-COII. The relative quantitation of OXPHOS subunit and SOD2 expression represents the mean ± SD of at least three experiments. Signal intensity for each antibody was normalized to the mean of high (OV3, OV4, and OV5) and low (OV8, SK3, and IGR1) OXPHOS expressing cell lines and Ponceau S staining (**Figures S2, S3**). **(B)** mtROS and mitochondrial mass were determined by MitoSOX-Red (MitoSOX) and MitoTracker-Red (MitoTracker) stains, respectively, using flow cytometry of live ovarian cancer cell lines. MitoSOX/MitoTracker ratio reflects mtROS formation per functional mitochondrion for each cell line.

Tumor-initiating cells undergo hypoxic conditions as they form spheroids and malignant ascites in the peritoneal cavity, causing changes in mitochondrial morphology and ROS levels during the progression of ovarian cancer (41, 47). mtROS produced as byproducts of OXPHOS when electrons leak from complexes I and III play a critical role in regulating a wide variety of cellular signaling pathways, including stabilization of hypoxia-inducible factor 1 alpha (HIF1α) in cancer (11, 27, 48–50). Therefore, it is critical to correlate OXPHOS status, mitochondrial biogenesis, and mtROS generation in ovarian cancer cell lines. For this purpose, we acquired the NCI-60 ovarian cancer cell line panel containing OVCAR-3, OVCAR-4, OVCAR-5, OVCAR-8, SKOV-3, and IGROV-1 cells derived from adenocarcinomas and peritoneal ascites (**Table S2**). Among these cell lines, OVCAR-3 and OVCAR-4 are the closest cell line models to HGSOC by comparing the genomic profiles (51, 52). The cells originated from ascites form aggressive peritoneal tumors and malignant ascites in animal models (**Table S2**) (53–55). In fact, the diversity of these cell lines might provide distinct mitochondrial characteristics and allow us to evaluate mitochondrial biogenesis and mtROS in these cell lines and compare it to HGSOC tumors. We first performed the immunoblotting analyses of cell lysates using the OXPHOS antibody cocktail as described in **Figure 1A**. The steady-state expression of OXPHOS subunits was relatively higher in OVCAR-3, OVCAR-4, and OVCAR-5 cells than that of the OVCAR-8, SKOV-3, and IGROV-1 cell lines (**Figure 4A**). These observations are all in agreement with high- and low-OXPHOS ovarian cancer cell line classification determined by Gentric et al. (7). Expressions of both mt-encoded, MT-COII (shown by a red arrow) and nuclear-encoded subunits, UQCRC2, NDUFB8, and COX4, were highly modulated between the two groups, confirming the high- and low-OXPHOS capacities in these cell lines (**Figure 4A**). Additionally, the increase in Mn-superoxide dismutase, SOD2, expression was more prominent in cells with high-OXPHOS capacity except the SKOV-3 cells (**Figure 4A**). The cells with higher OXPHOS subunit expression (**Figure 4A**) are suggested to be more sensitive to chemotherapy relative to the low OXPHOS expressing cells (7). As summarized in **Table S2**, some of the cell lines, specifically OVCAR-8 and SKOV-3, with reduced OXPHOS subunit expression and mitochondrial mass cause subcutaneous and intraperitoneal tumor formation in mice xenografts (54, 55).

Above, we demonstrated that the low-OXPHOS subunit expressing cell lines, OVCAR-8 and SKOV-3, are derived from highly malignant and chemo-resistant peritoneal ascites (**Figure 4A**) (**Table S2**). Due to the varying levels of OXPHOS subunit expression, one may speculate that the different mtROS levels adapted these cell lines to hypoxic conditions and survival in the peritoneal cavity. To correlate the OXPHOS subunit expression to mtROS generation, we performed flow cytometry analyses using MitoSOX-Red as well as the MitoTracker-Red staining of live ovarian cancer cell lines. The MitoSOX-Red and MitoTracker-Red ratios allowed us to determine the generation of mtROS per functional mitochondrion in these cell lines. This ratio was higher for OVCAR-8 cells relative to the other cell lines, specifically OVCAR-3, OVCAR-4, and OVCAR-5 cells (**Figure 4B**). In other words, lower MitoSOX/MitoTracker ratio in these cells with higher OXPHOS subunit expression could be either due to the increased mitochondrial mass or mtROS scavenging capacity. The reduced OXPHOS subunit expression was in agreement with the increased mtROS generation, specifically for OVCAR-8 and SKOV3 cells (**Figures 4A, B**). Although the reduced expression of SOD2 explains the increased mtROS generation in OVCAR-8 cells, the high SOD2 protein expression was not sufficient to suppress mtROS generation in SKOV-3 cells (**Figures 4A, B**). These cell lines were highly proliferative and resistant to cisplatin treatments [data not shown and (7)]. Again, the OVCAR-8 and SKOV3 cell lines are known to develop subcutaneous and intraperitoneal tumors in mice (54, 55); the reduced OXPHOS subunit expression can be associated with increased mtROS generation and resistance to chemotherapy, as also suggested by Gentric et al. (6, 23). Our observations and findings from other laboratories indicate that the remodeling of OXPHOS and mtROS generation could be highly informative in explaining tumor aggressiveness, metastasis to the peritoneal cavity, and recurrence in ovarian cancer (3, 18, 34, 41, 56).

### 3.4 Mitochondrial biogenesis is altered in ovarian cancer cell lines

**Figure 5:**
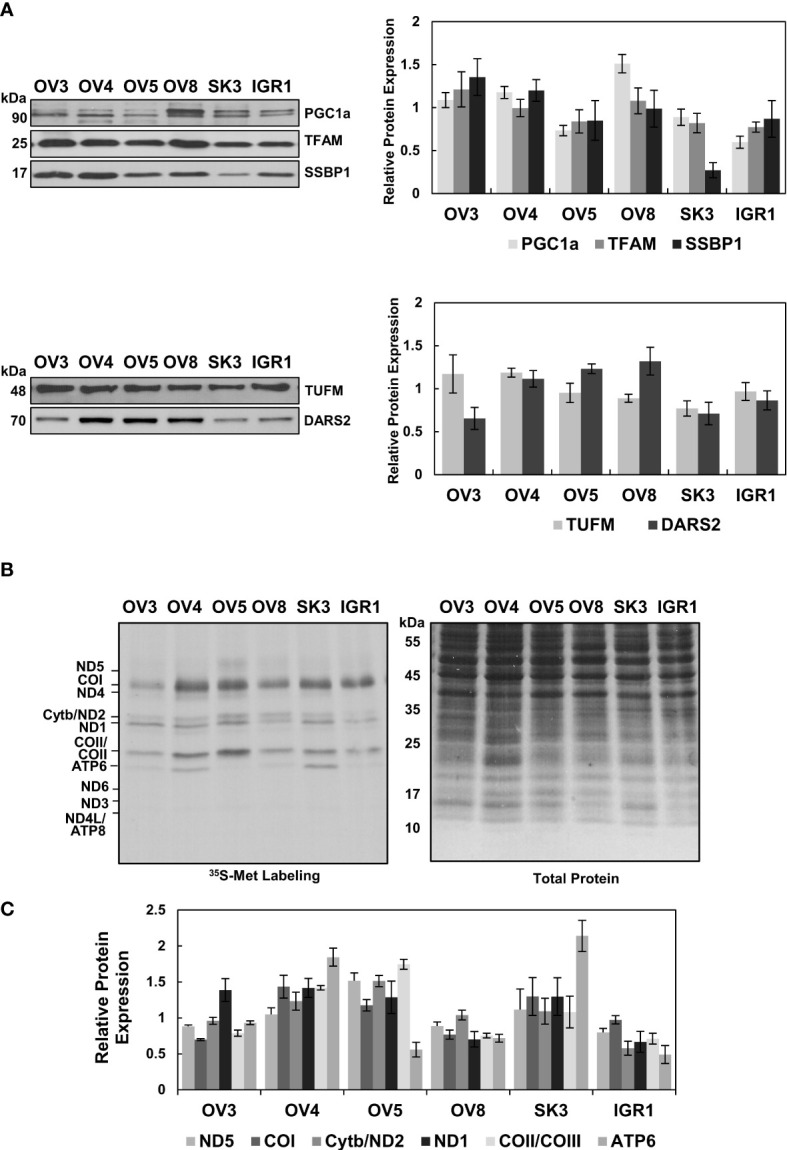
Evaluation of Mitochondrial Biogenesis in Ovarian Cancer Cell Lines. **(A)** Mitochondrial transcription and replication-related proteins, PGC1a, TFAM, and SSBP1, and **(B)** mitochondrial translation-related proteins, TUFM and DARS2, were detected by immunoblotting analyses of ovarian cancer cell lines described in **Figure 4A**. Ponceau S staining ensured equal protein loading Results represent the mean ± SD of at least three experiments. **(C)** Mitochondrial translation is determined by ^35^S-Met pulse labeling of 13 mt-encoded OXPHOS subunits in ovarian cancer cell lines. The pulse-labeled protein lysates (30 μg) were separated on 13% SDS-PAGE and 13 mt-encoded subunits, ND1-ND6 (complex I), Cyt b (complex III), COI-COIII (complex IV), and ATP6 and ATP8 (complex V), were labeled on the autoradiography of the gel. Total protein loading was visualized by Coomassie Blue. The relative quantitation of *de novo* synthesized subunits, ND5, COI, Cyt b/ND2, ND1, COII/COIII, and ATP6, was determined from at least three experiments, with the mean ± SD displayed. The signal intensity of each protein band was normalized to the mean of high (OV3, OV4, and OV5) or low (OV8, SK3, and IGR1) OXPHOS expressing cell lines.

Similar to our observations with HGSOC biopsies, the substantial change in the steady-state MT-COII expression implied the modulation of mitochondrial biogenesis in some of the ovarian cancer cell lines (**Figure 4A**). To further investigate this phenomenon, immunoblotting analyses of ovarian cancer cell lysates were carried out using PGC1α, TFAM, SSBP1, TUFM, and DARS2 antibodies. Expression of the major mitochondrial transcription factors, PGC1α and TFAM, and SSBP1 were relatively similar in these cell lines, with some exceptions (**Figure 5A**). Reduced SSBP1 protein expression was noteworthy in SKOV3 cells (**Figure 5A**). Like TFAM expression, variation in translation elongation factor TUFM expressions was negligible in these cell lines (**Figure 5B**). Another translation-related protein DARS2 expression was higher in cell lines with high OXPHOS capacity (**Figure 5B**). The lower DARS2 expression in OVCAR-3 cell lines is noteworthy and possibly attributable to the reduced aspartate levels in HGSOC (57). In general, the lack of any significant trend in the data suggests mitochondrial biogenesis could be regulated at different stages to modulate OXPHOS subunit expression in these cell lines.

One of the best methods to explore the functionality of mitochondrial biogenesis *in situ* is to perform pulse-labeling of *de novo* synthesized mitochondrial proteins in the presence of [^35^S]-Met. For this purpose, pulse labeling of ovarian cancer cell lines was carried out using cells grown to 70% confluency in regular media, as described previously (58). The *de novo* synthesized thirteen mt-encoded subunits were expressed by autoradiography after adding emetine in the media containing [^35^S]-Met and normalized to the total protein loading stained with Coomassie Blue. (**Figure 5C**). The expression of [^35^S]-Met-labeled subunits was clearly higher in OVCAR-4, OVCAR-5, and SKOV-3 cells relative to OVCAR-3, OVCAR-8, and IGROV-1 cells. Therefore, the reduced *de novo* protein synthesis observed in OVCAR-8 and IGROV-1 cell lines could be caused by mitochondrial translation and transcription defects (**Figure 5C**).

The steady-state expression of nuclear and mt-encoded OXPHOS subunits (**Figure 4A**) supported the results obtained with the *de novo* expression of 13 mt-encoded subunits for OVCAR-4, OVCAR-5, OVCAR-8, and IGROV-1 cells, with some exceptions (**Figure 5C**). The discrepancies observed between the steady-state and *de novo* expression of subunits in OVCAR-3 and SKOV-3 cell lines was conceivably caused by a difference in their proliferation rates (data not shown). For example, although the OVCAR-3 cells had higher steady-state subunit expression levels, the *de novo* subunit expression of OXPHOS subunits was lower than that of OVCAR-4 and OVCAR-5 cells. On the contrary, the SKOV-3 cell line had shown relatively high *de novo* OXPHOS subunit expression, possibly due to the proliferation rates of these cell lines.

Among the cells with low OXPHOS subunit expression, OVCAR-8, and SKOV-3, have higher invasion and metastatic capabilities supported by glycolytic energy metabolism rather than OXPHOS compared to the cell lines with higher OXPHOS capacity and resistance to cisplatin-induced apoptosis (**Figure 4A** and **Table S2**) (7, 27). The changes in *de novo* protein synthesis and the expression of mitochondrial translation components in these cell lines could also be correlated to alterations observed in HGSOC tumor biopsies.

## 4 Conclusions and future directions

HGSOC is one of the most common ovarian cancer subtypes and remains one of the deadliest cancers due to its high metastatic capacity and development of resistance to chemotherapy. With the metabolic heterogeneity of tumors in mind, one of the controversies that need to be resolved is the contribution of OXPHOS to aggressiveness and the development of chemoresistance and recurrence in ovarian cancer. One proposed mechanism for the high metastatic capacity and chemoresistance in ovarian cancer is the formation of spheroids from primary tumors with metabolic flexibility adapted to hypoxia in an ascitic environment. The increased metabolic flexibility of spheroids caused by modulation of mitochondrial function and morphology allows them to disseminate or reattach in the peritoneal cavity (4, 41, 47, 53, 59).

In this study, we investigated the role of mitochondrial biogenesis in the modulation of mitochondrial energy metabolism in HGSOC biopsies and ovarian cancer cell lines derived from adenocarcinomas and peritoneal ascites. The mitochondrial energy metabolism is evaluated by OXPHOS subunit expression belonging to the electron transport chain complexes (complexes I-IV) and ATP synthase (complex V). The primary and significant changes were observed in complex II and IV subunits, SDHB and MT-COII, respectively, in HGSOC biopsies (**Figures 1A, B**). In MS-based proteomics studies performed by McDermott et al., the overall change was insignificant except for a reduction in NDUFS2 expression (**Figure 1C**) (45). On the other hand, the difference and increase between the low- and high-OXPHOS expressing HGSOC biopsies was significant for all the subunits (**Figure 1D**) (7). The heterogeneity and the increased OXPHOS subunit expression in most of the HGSOC biopsies reported by Gentric et al. (7) strongly agree with the bimodal distribution observed in our analyses, specifically for MT-COII expression (**Figure 1**). Interestingly, the high-OXPHOS tumors have shown an increased response to conventional chemotherapy and are associated with better prognosis in HGSOC patients (7).

Expression of the mt-encoded OXPHOS subunit quantified in our analysis, MT-COII, depends on mitochondrial biogenesis; thus, the modulation of transcription and translation machineries in mitochondria is essential. Probing normal and HGSOC biopsies for the expression of PGC1α, TFAM, SSBP1, TUFM, and DARS2 allowed us to demonstrate the correlation between the changes in OXPHOS subunit expression and mitochondrial biogenesis for the first time (**Figures 1**–**3**). The strong agreement between our findings on mitochondrial biogenesis and the data mining analyses of MS-based proteomics studies allowed us to suggest that the remodeling of energy metabolism or metabolic flexibility in HGSOC is regulated by mitochondrial biogenesis (**Figures 1**–**3**) (7, 45). Since metabolic flexibility is one of the determinants of the survival of tumor cells in the peritoneal cavity, deciphering mitochondrial biogenesis and its role in the remodeling of energy metabolism is crucial for better prognosis in HGSOC patients.

The mechanism behind the chemotherapy resistance and recurrence is still an unresolved issue in HGSOC (60, 61). The role of mitochondrial function and oxidative stress in chemoresistance and metastatic capacity is under investigation by many groups using ovarian cancer cell line models (5, 7, 27, 41, 47, 53). Here, we aimed to correlate mitochondrial biogenesis and mtROS generation to OXPHOS status in commonly used NCI-60 ovarian cancer cell line panel, including highly metastatic and chemoresistant cell lines listed in **Table S2**. Two of the cell lines with lower OXPHOS subunit expression, OVCAR-8, and SKOV-3 (**Figure 4A**), are the more aggressive cell lines forming subcutaneous and intraperitoneal tumors in mice xenografts (**Table S2**) (54, 55). Gentric et al. have suggested that ovarian cancer cell lines, including OVCAR-8 and SKOV-3, and HGSOC tumors with low-OXPHOS subunit expression have decreased chemosensitivity to platinum-based treatments due to reduced oxidative stress and ferroptosis (7). Modulating oxidative stress and ROS generation is proposed as one of the mechanisms behind the cis-platin induced cell death (3, 5, 27, 56, 62); however, it might not be possible to modulate mtROS generation in cells or tumors with low-OXPHOS. Although the overall cellular ROS generation is lower in low-OXPHOS cells (7), we found that the mtROS per functional mitochondrion was higher in OVCAR-8 cells, which is known for its resistance to cis-platin cell death relative to the high-OXPHOS cell lines (**Figure 4B**). It is conceivable that the low-OXPHOS cells or HGSOC tumors already have leaky electron transport chain generating high mtROS levels due to reduced mitochondrial biogenesis and SOD2 levels (**Figure 4A**). The decreased SOD2 levels or antioxidant capacity of ovarian cancer cells with low-OXPHOS, such as OVCAR-8, is not sufficient to scavenge mtROS generated in these cells (**Figure 4**). One possible mechanism for resistance to platinum-based treatments in OVCAR-8 cells could be due to the low SOD2 levels and increased mtROS generation. Dual role of SOD2 expression has been suggested in ovarian cancer as tumor suppressor and protumoregenic factor (53, 63–65); therefore, antioxidant capacity of HGSOC need to be critically evaluated in future studies.

As discussed above, the association between the OXPHOS subunit expression and mitochondrial biogenesis is clearly shown in HGSOC tumor biopsies (**Figures 1**–**3**). This correlation was not clear with the ovarian cancer cell lines with low-OXPHOS subunit expression; specifically, the OVCAR-8 cells had normal levels and, in some cases, higher levels of PGC1α, TFAM, TUFM, and DARS2 protein expression (**Figures 5A, B**). On the other hand, the *de novo* synthesis of mt-encoded proteins determined by the ^35^[S]-Met pulse labeling experiments was consistent with the MT-COII expression detected by immunoblotting analyses in some of the cell lines (**Figures 4A**, **5C**). The *de novo* protein synthesis data obtained with the ovarian cancer cells suggests mitochondrial biogenesis, at both transcription and translation levels, remodels the mitochondrial energy metabolism and possibly mtROS generation in ovarian cancer.

Changes in mitochondrial mRNA and biogenesis related transcript levels are previously utilized in predicting overall survival and tumor progression and suggested as possible prognostic biomarkers in HGSOC (33–35). In this study, we validated these predictions at the protein expression levels and have shown the role of mitochondrial biogenesis in metabolic remodeling of HGSOC tumors and ovarian cancer cell lines derived from ascites. One of the major limitations of our study is the absence of normal ovarian and true HGSOC cell lines for a better comparison of changes in OXPHOS and mitochondrial biogenesis. The role of mitochondria in metabolic remodeling of HGSOC is now better-understood (2, 5, 7, 18, 66, 67); however, the role of mitochondrial biogenesis in formation of peritoneal spheroids and ascites as well as mtROS generation need to be investigated further to improve efficacy of current chemotherapy options in HGSOC treatment.

## Data availability statement

The original contributions presented in the study are included in the article/**Supplementary materials**, further inquiries can be directed to the corresponding author/s.

## Ethics statement

Ethical review and approval were not required for the human de-identified biopsies used in this study in accordance with the local legislation and institutional requirements.

## Author contributions

ZK and EK designed the study. ZK and EK performed immunoblotting analyses of biopsies and ovarian cancer cell lines. EK performed ^35^S-Met pulse labeling assays. ZK and EK performed the data mining analysis of the CPTAC proteome and prepared the figures. EK and VS performed flow cytometry analyses of ovarian cancer cell lines. ZK, VS, NB, GR, and EK involved in manuscript writing and revision. All authors contributed to the article and approved the submitted version.
