# Supplementary material for: Evaluation of mitochondrial biogenesis and ROS generation in high-grade serous ovarian cancer
Source: Front Oncol. 2023 Mar 1;13:1129352. doi: 10.3389/fonc.2023.1129352 (PMC10014927; doi:10.3389/fonc.2023.1129352)
Supplement: Supplementary file 1 [file DataSheet_1.pdf]

SUPPLEMENTAL FIGURES

Figure S1. Comparison of Ponceau S staining of nitrocellulose membranes and β-tubulin probing used for normalization of protein loading in immunoblotting analyses of HGSOC biopsies. Approximately 20 μg of protein lysate from each normal and tumor biopsy was separated on 12% SDS-PAGE, and equal protein loading was normalized to β-tubulin signal and Ponceau S staining of the membranes. As shown by the arrow, albumin (ALB) protein expression largely varied between the adjacent normal and tumor tissues obtained from the same patient. For this reason, protein bands below the ALB band were used to normalize total protein loading for immunoblotting analyses. Only three nitrocellulose membranes were included as examples.

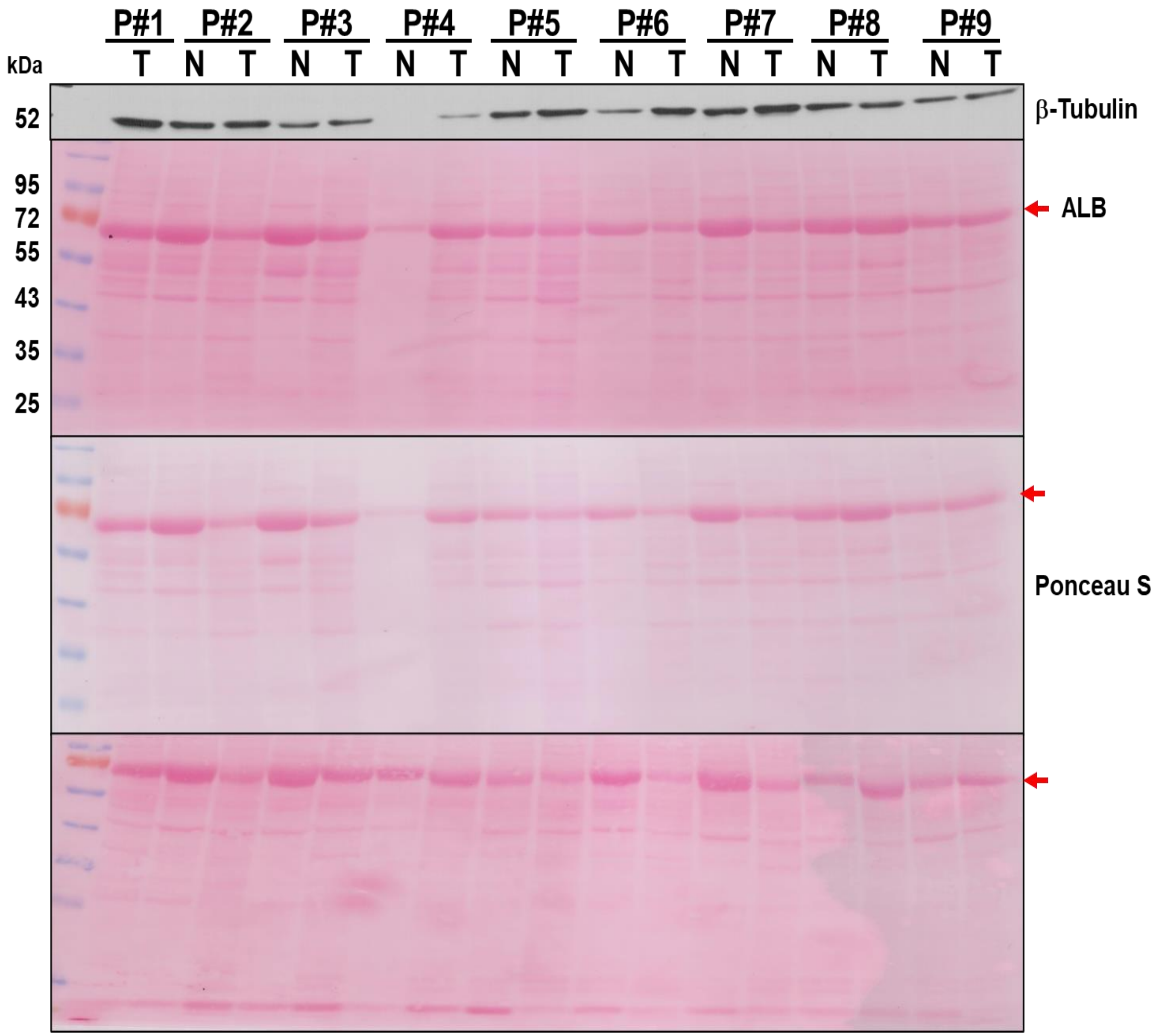

Figure S2. Examples of the nitrocellulose membranes stained with Ponceau S used in Figs. 4A and 5A, B to detect OXPHOS subunit and mitochondrial protein expression by immunoblotting. Approximately 20 µg of protein lysates obtained from ovarian cancer cell lines, OVCAR-3 (OV3), OVCAR-4 (OV4), OVCAR-5 (OV5), OVCAR-8 (OV8), SKOV-3 (SK3), and IGROV-1 (IGR1) were separated on 12% SDS-PAGE, and Ponceau S staining evaluated equal protein loading. Quantitation of OXPHOS subunit and mitochondrial protein expression (Figs. 4A, 5A, and 5B) were normalized to total protein loading detected by Ponceau S-stained membranes.

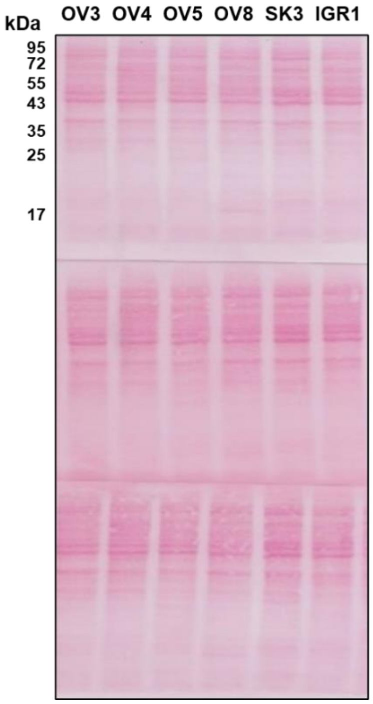

Figure S3. Immunoblotting analysis of SOD2 expression in ovarian cancer cell lines. SOD2 (Mn-superoxide dismutase detected by immunoblotting as described in Materials and Methods and Fig. 4A. To normalize the protein amounts used in SOD2 detection, approximately 20 µg of protein lysates obtained from ovarian cancer cell lines, OVCAR-3 (OV3), OVCAR-4 (OV4), OVCAR-5 (OV5), OVCAR-8 (OV8), SKOV-3 (SK3), and IGROV-1 (IGR1) were separated on 12% SDS-PAGE, and Ponceau S staining evaluated equal protein loading. **B)** Relative SOD2 protein expression detected by immunoblotting (Fig. 4A) was quantified and normalized to Ponceau S-stained membranes.

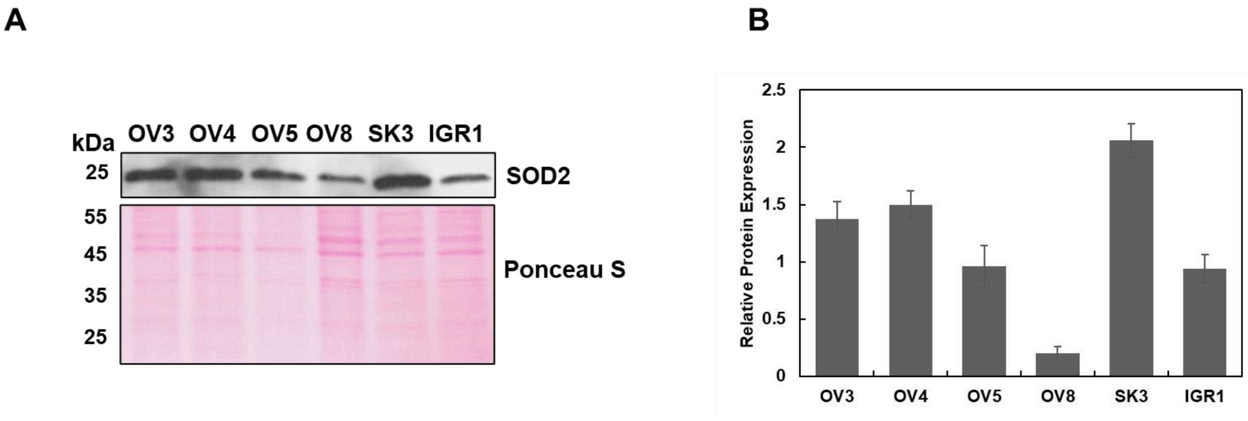
A
B
kDa
OV3
OV4
OV5
OV8
SK3
IGR1
25
SOD2
55
45
35
25
Ponceau S
Relative Protein Expression
2.5
2
1.5
1
0.5
0
