# Supplementary material for: Evaluation of mitochondrial biogenesis and ROS generation in high-grade serous ovarian cancer
Source: Front Oncol. 2023 Mar 1;13:1129352. doi: 10.3389/fonc.2023.1129352 (PMC10014927; doi:10.3389/fonc.2023.1129352)
Supplement: Supplementary file 2 [file DataSheet_2.pdf]

SUPPLEMENTAL TABLES

Table S1. The Tumor Characteristics of Ovarian Carcinoma Biopsies. Fifteen de-identified ovarian tumors and normal tissue biopsies were removed by surgical excision from patients treated at the Marshall University Edwards Comprehensive Cancer Center, Huntington, WV. Ovarian cancer subtypes were determined by immunohistochemistry, immunofluorescence, and fluorescence in situ hybridization techniques by the Edwards Comprehensive Cancer Center.

**Patient #**	**BMI**	**Stage**	**Diagnosis**
1	39.1	pT2cN0	High grade serous carcinoma
2	27.1	pT1NX	High grade serous carcinoma
3	26.1	pT3cNX	High grade serous carcinoma
4	32.1	pT3bNX	High grade serous carcinoma
5	24.7	pT3cN1a	High grade serous carcinoma
6	37.3	pT3aN2a	High grade serous carcinoma
7	20.8	pT1bNX	High grade serous carcinoma
8	42.8	pT3aN1	Low grade serous carcinoma
9	34.9	pTb1NX	Serous borderline carcinoma
10	31.7	ypT3cN1b	High grade serous carcinoma
11	23.89	ypT3cNX	High grade serous carcinoma
12	16.22	pT3cNX	High grade serous carcinoma
13	30.6	pT3bNX	High grade serous carcinoma
14	29.84	pT3cNX	Low grade serous carcinoma
15	24.15	pT3cNX	Low grade serous carcinoma

pT: Tumor stage
pN: Metastasis
y: Previously treated

Table S2. Tumor characteristics of ovarian cancer cell lines in NCI-60 panel and formation of subcutaneous and intraperitoneal tumors in mice.

**Name**	**Histology**	**Tissue Site**	**p53 Status**	**Sub.**	**Intra.**
OVCAR-3	Serous G2	Ascites	Mutant	0/4	1/4
OVCAR-4	Adenoca. G2	Ascites	WT	0/4	1/4
OVCAR-5	Adenoca. G1	N/A	Absent	4/4	2/4
OVCAR-8	Adenoca. G3	N/A	Del. (Y126-K132)	4/4	2/3
SKOV-3	Serous	Ascites	Absent	4/4	3/4
IGROV-1	Serous G3	Primary	Y126C	4/4	0/3

The number of mice that formed subcutaneous and intraperitoneal tumors per number of mice tested are taken from Mitra *et al.* [1] and Hernandez *et al.* [2] and references therein. OVCAR-5 cell line is also reported as being gastrointestinal using gene expression compositional assignments [3]; however, NCI did not confirm this report. Abbreviations: Adeno: adenocarcinoma, Sub: subcutaneous, Intra; intraperitoneal, WT: wild type, Del: deletion, N/A: not available. In the original nomenclature, SKOV-3 and IGROV-1 cells were abbreviated as SK-OV-3 and IGR-OV1, respectively.

1. Mitra, A.K., et al., *In vivo tumor growth of high-grade serous ovarian cancer cell lines.* Gynecol Oncol, 2015. **138**(2): p. 372-7.
2. Hernandez, L., et al., *Characterization of ovarian cancer cell lines as in vivo models for preclinical studies.* Gynecol Oncol, 2016. **142**(2): p. 332-40.
